# A Homeostatic Model of Neuronal Firing Governed by Feedback Signals from the Extracellular Matrix

**DOI:** 10.1371/journal.pone.0041646

**Published:** 2012-07-27

**Authors:** Victor Kazantsev, Susan Gordleeva, Sergey Stasenko, Alexander Dityatev

**Affiliations:** 1 Laboratory of Nonlinear Dynamics of Living Systems, Institute of Applied Physics of Russian Academy of Science, Nizhny Novgorod, Russia; 2 Laboratory for Brain Extracellular Matrix Research, University of Nizhny Novgorod, Nizhny Novgorod, Russia; 3 Department of Neuroscience and Brain Technologies, Istituto Italiano di Tecnologia, Genova, Italy; 4 Molecular Neuroplasticity Group, DZNE, German Center for Neurodegenerative Diseases, Magdeburg, Germany; National Center for Scientific Research Demokritos, Greece

## Abstract

Molecules of the extracellular matrix (ECM) can modulate the efficacy of synaptic transmission and neuronal excitability. These mechanisms are crucial for the homeostatic regulation of neuronal firing over extended timescales. In this study, we introduce a simple mathematical model of neuronal spiking balanced by the influence of the ECM. We consider a neuron receiving random synaptic input in the form of Poisson spike trains and the ECM, which is modeled by a phenomenological variable involved in two feedback mechanisms. One feedback mechanism scales the values of the input synaptic conductance to compensate for changes in firing rate. The second feedback accounts for slow fluctuations of the excitation threshold and depends on the ECM concentration. We show that the ECM-mediated feedback acts as a robust mechanism to provide a homeostatic adjustment of the average firing rate. Interestingly, the activation of feedback mechanisms may lead to a bistability in which two different stable levels of average firing rates can coexist in a spiking network. We discuss the mechanisms of the bistability and how they may be related to memory function.

## Introduction

Recent studies have uncovered multiple mechanisms by which extracellular matrix (ECM) molecules regulate various aspects of synaptic activity and highlighted a link between the ECM and learning and memory [1]. In addition to synaptic plasticity, which can be rapidly induced in response to sensory stimuli and helps adaptation to the environment, homeostatic forms of plasticity operate on a longer timescale and help preserve neural cells by preventing the pathological hypo- or hyper-excitation of neurons, which can lead to neural dysfunction and cell death. For instance, homeostatic regulation of synaptic strength, termed synaptic scaling, allows neurons to maintain their firing rates within a certain range despite perturbations (such as changes in sensory inputs) imposed on the network [2], [3]. In response to a prolonged blockade of action potentials by tetrodotoxin, all excitatory synapses on pyramidal cells are equally ‘scaled up’, due to an increase of postsynaptic AMPA receptor density that has been detected by analysis of the amplitude distribution of miniature excitatory postsynaptic currents (mEPSCs) [2], [3]. The mechanism underlying synaptic scaling involves glia-derived tumor necrosis factor (TNF) [4], [5], which upregulates the expression of β3 integrins at the postsynaptic cell surface. Signaling via these integrins inhibits the small GTPase Rap1 and decreases endocytosis of synaptic GluA2 glutamate receptors, thereby increasing synaptic strength.

Another mechanism of homeostatic regulation involves chondroitin sulfate proteoglycans (CSPGs), which are proteins covalently linked to chondroitin sulfate glycosaminoglycans with a complex pattern of sulfate groups [6]. CSPGs are enriched in the ECM associated with the perineuronal nets covering, for instance, fast-spiking interneurons. Formation of this ECM form requires neuronal firing and the activity of L-type voltage-dependent Ca2+ channels and Ca2+ permeable GluA2-lacking glutamate receptors [7]. Acute removal of chondroitin sulfates with chondroitinase ABC treatment elevates the intrinsic excitability of perisomatic hippocampal interneurons [7]. Thus, it appears that during development, neuronal activity drives the formation of the ECM that, in turn, inhibits neuronal excitability.

Apart from the activity-dependent expression and secretion of ECM molecules into the extracellular space, another important factor controlling the ECM is the activity of extracellular proteases. Because this study is the first modeling study of the homeostatic function of the ECM, we introduced a minimal set of phenomenological variables to describe the influence of activity-dependent accumulation of ECM, protease and ECM receptor activity in the context of synaptic scaling and ECM-dependent changes in excitability.

**Figure 1:**
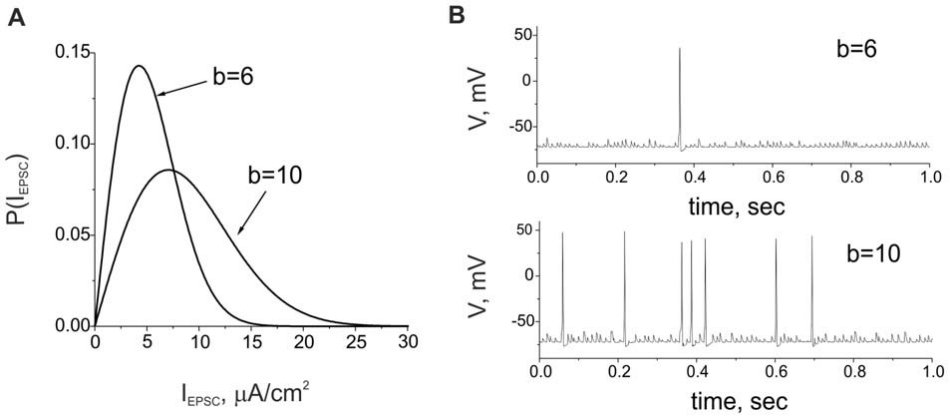
Neuron response to excitatory stimulation. **A.** Probability densities for EPSCs amplitudes according to Eq. (5) for *b = *6 and *b* = 10. **B.** Oscillations of membrane potential in Eqs. (1)–(5) for different strengths of synaptic input.

In modeling, any regulation mechanism implies the existence of a feedback loop tuning the neuron toward a stable state. For synaptic regulation, many phenomenological and biophysical models of synaptic plasticity have been proposed [8]–[10]. Generally, feedback that can be mediated by different molecular cascades leads to changes in the synaptic weights that tune the neuron to increase (facilitation) or decrease (depression) its firing rate. These models represent dynamical systems in which the synaptic weight is considered to be a function of firing rate (rate coding) or the relative time instances of pre- and postsynaptic spike occurrence (spike timing-dependent plasticity). Synaptic changes in these models occur at timescales of milliseconds and seconds and reflect the characteristic times of information processing in neuronal networks. Another feedback may be organized in the frame of the tripartite synapse concept, which involves astrocyte activity in regulating synaptic transmission efficacy [11]. Many studies have shown that astrocyte-mediated feedback can influence synaptic function at the timescale of dozens of seconds, which reflects the timescale of calcium signals in astrocytes [12], [13].

**Figure 2:**
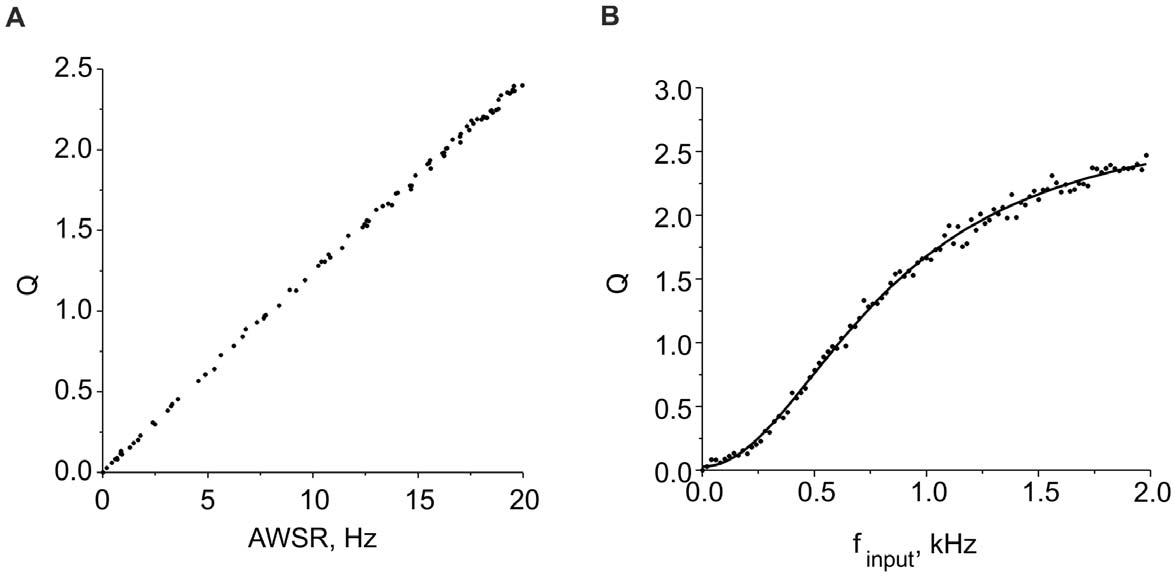
Characteristics of average activity. **A.** Average activity values versus average spiking rate (AWSR) calculated in the model Eqs. (1)–(6) for different input frequencies over the time window *T = 2 min*. **B.** Average activity *Q* versus input frequency. The solid curve shows the logistic curve (Eq. (8)) fitting the data. Parameter values: *I_th_ = 4.5 µA/cm^2^, b = 6, k_q_* = 0.01, *α_q_* = 0.0001 *mses^−1^*, and *β_q_* = 0.01 *msec^−1^*.

In the tetrapartite synapse that involves the ECM [14], the timescale of ECM changes is expected to last for hours and days and reflect the cumulative integration of neuronal firing that drives activity-dependent synthesis and the secretion of ECM molecules. The consequences of such changes have not been incorporated into existing synaptic regulation models and are not yet fully understood. In the proposed model, we use the concept of activity-dependent *activation functions* typically used for phenomenological descriptions of neural excitability (e.g., gating functions for voltage-dependent channels in the Hodgkin-Huxley formalism [15]). Based on experimental observations, we choose certain shapes of the activation functions and analyzed the computational consequences of ECM-mediated feedback. The feedback is organized as two circuits changing neuronal excitability and the weights of synaptic inputs. We analyze the existence and stability of steady state solutions and predict that the ECM-mediated feedback may provide a robust mechanism of homeostatic regulation over long timescales. We also show the appearance of bistability as an intrinsic feature of the regulation scheme involving the ECM.

**Figure 3:**
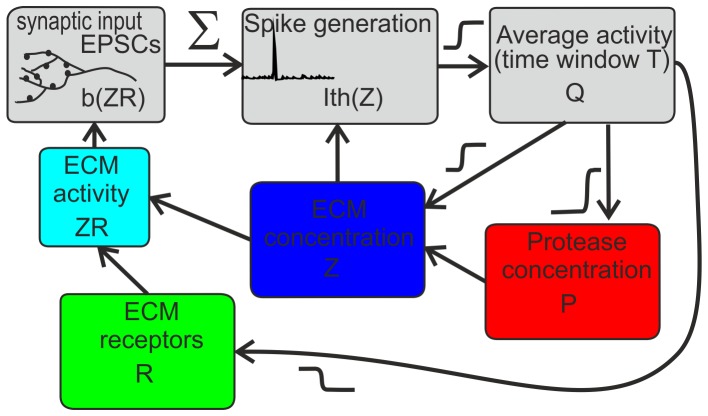
Schematic illustration of ECM-induced homeostatic regulation of average activity (see details in the main text).

## Methods

### 1.1. Membrane Excitability

We take the widely used Hodgkin-Huxley equations to model spike generation in a neuronal cell (for example, see [15]). The membrane potential evolves according to the following current balance equation:


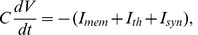
(1)

where *I_mem_ = I_Na_+I_K_+I_leak_* is the sum of the transmembrane currents responsible for spike generation:


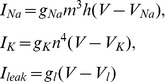
(2)

*I_th_* is an applied current regulating effective spike excitation threshold. Higher values of *I_th_* results in hyperpolarization of the neuron, hence, larger input is needed to reach the threshold of spike generation, e.g. the effective excitation threshold is increased. *I_syn_* is the total synaptic input to the neuron. The gating variables in Eqs. (2) evolve according to the following equations:


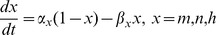
(3)

Parameters and nonlinear functions for gating variables in (2)–(3) are taken as in the classical Hodgkin-Huxley equations provided elsewhere [15].

**Figure 4:**
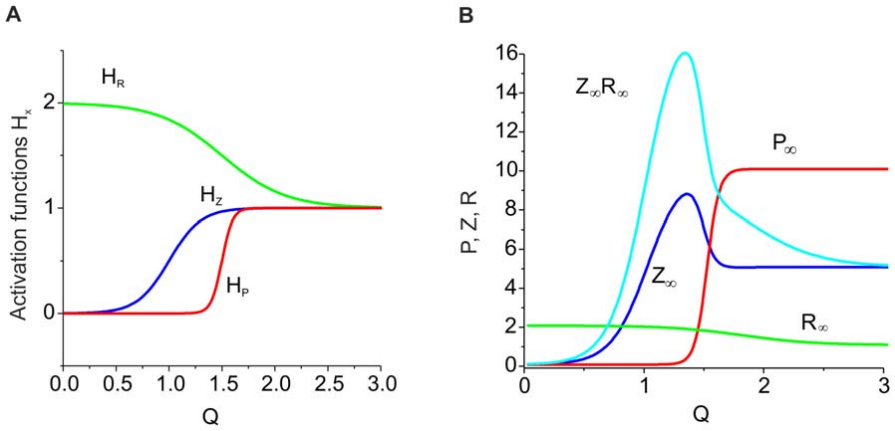
Activation kinetics for the concentrations of ECM molecules, proteases and ECM receptors, respectively. **A.** Activation functions for model (9). Parameter values: *z_0_ = p_0_ = 0, z_1_ = p_1_ = 1, r_0_ = 2, r_1_ = 1,θ_z_ = 1 k_z_ = 0.15, θ_p_ = 1.5, k_p_ = 0.05, θ_r_ = 1.8,* and *k_r_ = 0.1*. **B.** Steady state distribution profiles for *Z_∞_*, *P_∞_*, and *R_∞_* are shown by solid curves. The dashed curve illustrates the steady state values of *Z_∞_R_∞_* regulating the synaptic weights (see Eqs. (10)). Parameter values: *z_0_ = p_0_ = 0, z_1_ = p_1_ = 1, r_0_ = 2, r_1_ = 1,θ_z_ = 1,k_z_ = 0.15, θ_p_ = 1.5, k_p_ = 0.05, θ_r_ = 1.8, k_r_ = 0.1, α_z_* = 0.001 *msec^−1^*, *β_z_* = 0.01 *msec^−1^*, *α_p_* = 0.001 *msec^−1^*, *β_p_* = 0.01 *msec^−1^*, *α_r_* = 0.01 *msec^−1^*, *β_r_* = 0.01 *msec^−1^*, and *γ_P_* = 0.1.

### 1.2. Synaptic Input

As a member of neuronal network, a neuron receives a number of synaptic inputs. The input currents are associated with spiking events in presynaptic terminals. Considering spontaneous network activity, we assume that these events are generally uncorrelated and can therefore be expressed in the form of a Poisson spike train:


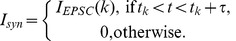
(4)

In this equation, *t_k_* accounts for the presynaptic spike occurrence times satisfying the Poisson distribution with a characteristic frequency *f_input_. τ ≈ 1* msec is the spike duration, and *I_EPSC_(k)* is the amplitude of the excitatory postsynaptic current evoked by transmitter release in response to presynaptic spikes. Following experimental observations [16], we assume that the amplitude of spontaneous EPSCs satisfy the probability distribution *P(I_EPSC_)*, shown in Fig. 1 A. We use the analytical expression for *P(I_EPSC_)* in the following form:


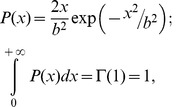
(5)

where *Γ* is the gamma function, and *b* is the scaling factor accounting for the effective strength of the synaptic input defined by Eqs. (4) and (5). Figure 1B illustrates the evolution of the membrane potential of the postsynaptic neuron driven by the synaptic input defined by Eqs. (4) and (5).

**Figure 5:**
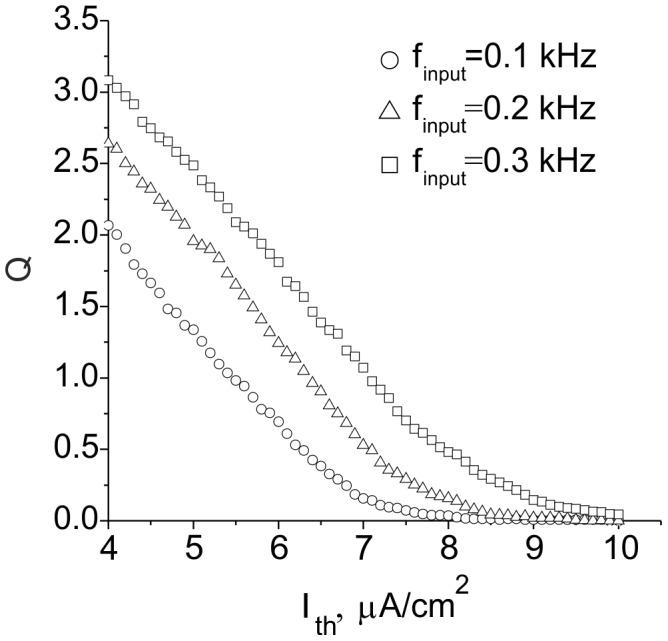
The dependence of average activity on threshold regulating parameter *I_th_* calculated from Eqs. (1)–(6) for different input frequencies. Parameter values: *b* = 10, *k_q_* = 0.01, *α_q_* = 0.0001 *msec^−1^*, and *β_q_* = 0.01 *msec^−1^*.

### 1.3. Average Activity Level

For the purpose of this study, we must characterize neuronal activity at notably long timescales compared with the duration of an action potential. Using Eqs. (1)–(5), which describe spike generation at the millisecond timescale *τ*, we introduce an average activity variable *Q* in the following form:


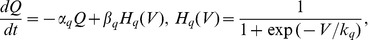
(6)

where *τ_q_* is a rate constant, *β_q_* is a scaling coefficient with *0<α_q_<β_q_*, and *k_q_* is the inverse slope of the activation function *H_q_(V)*, *k_q_<1*. The process defined by Eq. (6) represents a spike detector increasing variable *Q* by *ΔQ ≈ β_q_τ* for each successfully generated spike. Within the interval between spikes, *Q* decays exponentially until the next spike comes. Next, for *n*-spikes generated with average frequency *f = n/T*, *Q(n)* will converge to the limit as follows:


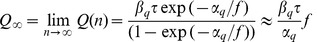
(7)

Eqs. (6) and (7) represent a technical analogue of a sliding window continuously accounting for the number of spikes during time interval *T* in the past. The contribution of spikes generated before time moment *t-T* is negligible because it decays exponentially with rate constant *α_q_*. Thus, the average activity *Q* is proportional to the average window spiking rate (AWSR) of the neuron, as illustrated in Fig. 2 A for a numerical simulation of Eqs. (1)–(6).

**Figure 6:**
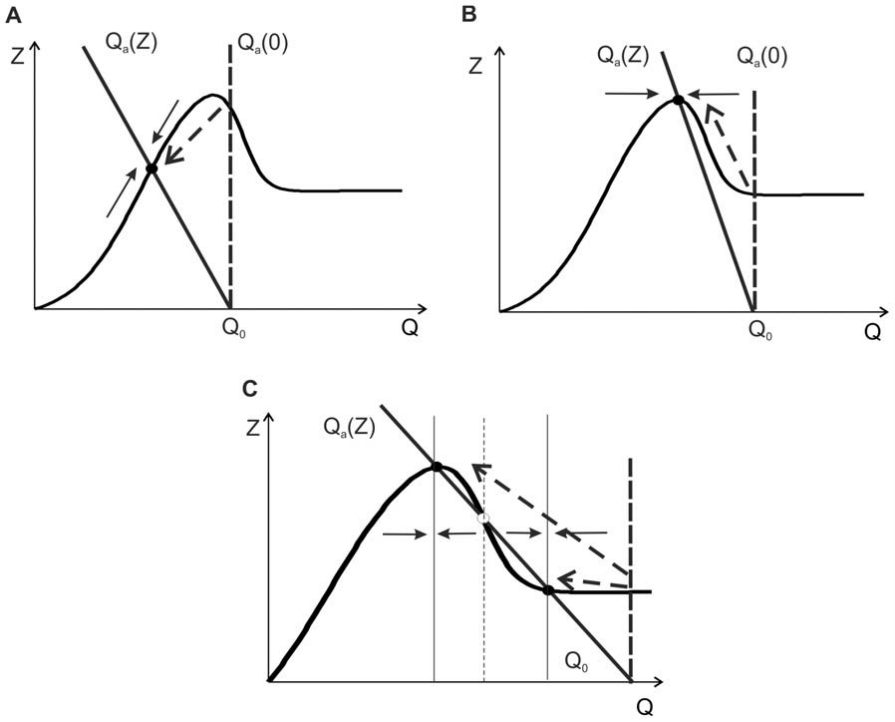
Schematic figures of equilibrium curves illustrated in the phase plane *(Z, Q)*. **A.** Single fixed point balance leading to a decrease of the ECM concentration. **B.** An increase of the ECM concentration due to deactivation of proteases for higher initial activity. **C.** Three fixed point (bistable) balance.

Note that in contrast to typically used sliding window techniques, the average activity *Q(t)* defined by Eq. (7) is a continuous function of time. Figure 2B illustrates the dependence of the average activity on the frequency of the input Poisson spike train, which can be fitted by a logistic curve of the following form:


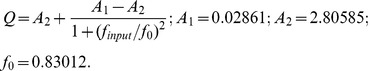
(8)

## Results

### 2.1. Neural Extracellular Matrix Feedback Model

To account for ECM-mediated homeostatic regulation, we adopted the following feedback circuit (Fig. 3), which describes the basic effects of the ECM influence on neuronal excitability and efficacy of synaptic transmission [1], [7], [16].

**Figure 7:**
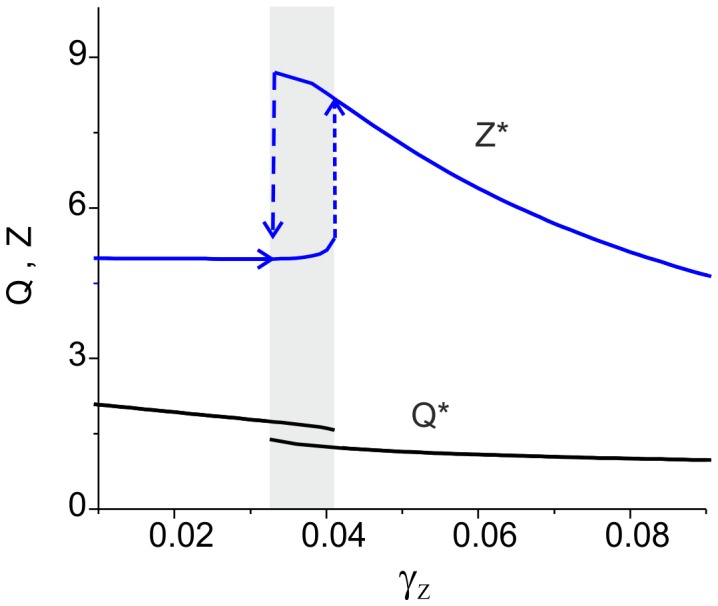
Dynamics of the ECM-protease regulation cascade depending on the feedback gain obtained in the simulation of Eqs. (9) and (14) for different initial conditions. The gray area illustrates bistability. Parameter values: *z_0_ = p_0_ = 0, z_1_ = p_1_ = 1, θ_z_ = 1,k_z_ = 0.15, θ_p_ = 1.5,k_p_ = 0.05, α_z_* = 0.001 *msec^−1^*, *β_z_* = 0.01 *msec^−1^*, *α_p_* = 0.001 *msec^−1^*, *β_p_* = 0.01 *msec^−1^*, *γ_P_ = 0.1, α_1_* = 0.0001 *msec^−1^*, *α_2_* = 0.001 *msec^−1^*, *I_0_ = 4.5, b_0_ = 6, Q_0_ = 2.23,* and *k_I_ = 0.6625*.

The EPSCs caused by spontaneous network firing are integrated in the dendritic tree and may lead to action potential (AP) generation if the effective excitation threshold controlled by *I_th_* is exceeded. Sequences of spontaneously generated APs over a long timescale are converted into the average firing rate *Q*, which is explicitly shown in the circuit in Fig. 3. Based on experimental observations [7], [17], we assume that neuronal firing stimulates ECM production and denote ECM concentration by *Z*. In turn, increasing concentrations of ECM molecules (e.g., chondroitin sulfate proteoglycans) increase the excitation threshold for action potential generation [7]. We account for this increase by the dependence of the AP effective excitation threshold on variable *Z, I_th_(Z)*. The ECM may slowly dissolve spontaneously and degrade due to the activation of extracellular proteases. Production, secretion and activity of numerous proteases are subsequently increased with higher activity levels [18].

**Figure 8:**
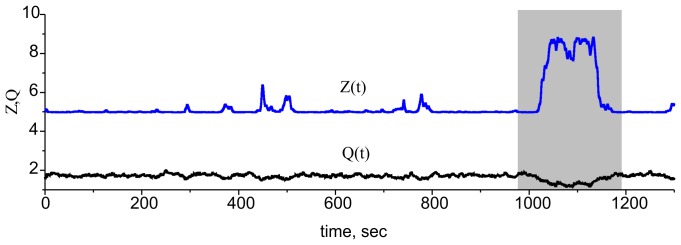
Simulation of ECM-proteases feedback regulating excitability in terms of the original model (1)–(6), (9), (10). Due to the integration of the Poisson input train, *Q* fluctuates near the *Q_∞_* value (see Eq. (7) in Methods). In the bistable mode, this fluctuation leads to spontaneous switches between high and low ECM concentrations and thus high and low activity values (time interval shown in gray). Parameter values: *z_0_ = p_0_ = 0, z_1_ = p_1_ = 1, θ_z_ = 1,k_z_ = 0.15, θ_p_ = 1.5,k_p_ = 0.05, k_q_* = 0.01, *γ_p_* = 0.1, *I_0_ = 4.5, b_0_ = 6, γ_Z_ = 0.0345, γ_ZR_ = 0.0, α_q_* = 0.0001 *msec^−1^*, *β_q_* = 0.01 *msec^−1^*, *α_z_* = 0.001 *msec^−1^*, *β_z_* = 0.01 *msec^−1^*, *α_p_* = 0.001 *msec^−1^*, *β_p_* = 0.01 *msec^−1^* and *f_input_ = 0.2 kHz*.

Other important factors are ECM receptors, such as integrins. We assume that their concentration *R* is also dependent on the average activity level. *R* is higher if the neuronal activity level decreases below some critical level [16]. Assuming that ECM production and ECM receptor expression are statistically uncorrelated processes, we describe the impact of ECM receptor activation by ECM molecules on the synaptic strength as a variable proportional to the product *ZR*. Therefore, the synaptic strength increases, i.e., there is a homeostatic synaptic up-scaling if the firing rate decreases below a critical level, and *R* concentration is upregulated.

**Figure 9:**
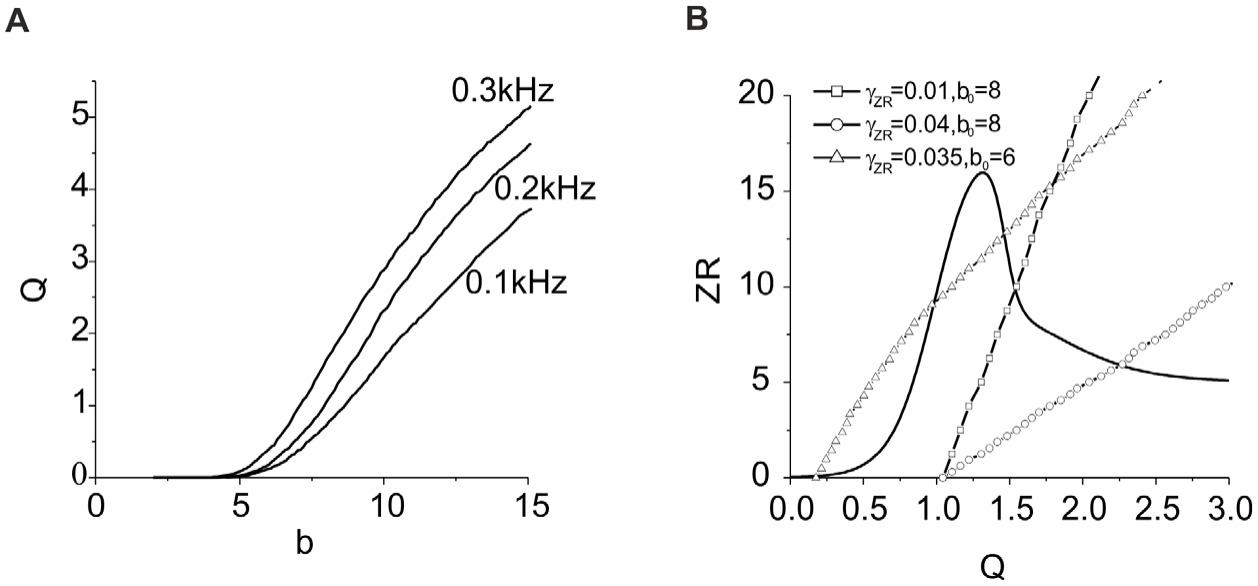
Characteristics of ECM receptor regulation cascade. **A.** Average dependence of activity on the strength of synaptic input for different Poisson frequencies. **B.** Equilibrium curves illustrating the solutions of Eqs. (17) for different ECM receptor feedback gains. Parameter values: *k_q_* = 0.01, *α_q_* = 0.0001 *msec^−1^*, *β_q_* = 0.01 *msec^−1^*, *I_0_ = 4.5, b_0_ = 6, γ_P_ = 0.01, γ_Z_ = 0.0,* and *γ_ZR_ = 0.01,0.04,0.035.*

Thus, the ECM regulation circuit comprises two basic feedback mechanisms.

- Activity dependent modulation of excitation threshold leading to fluctuations of the output firing rate. This feedback is bidirectional comprising two loops, including ECM production (decrease in excitability) and its inhibition by proteases (hence, increase in excitability).
- Change of the effective strength of synaptic inputs and thus the firing rate due to signaling via the ECM receptors. This change is also bidirectional; it may be potentiating or depressive, depending on the activity level.

### 2.2. Mathematical Model

Following the ECM feedback circuit, we propose the following mathematical model for ECM-induced homeostatic regulation of firing rates that comprises a set of three differential equations:


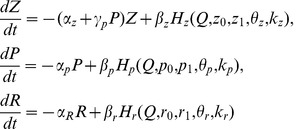
(9)

and two feedback functions that modulate neuronal dynamics (see Methods, Eqs. (1)–(6)):


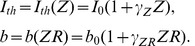
(10)

The activation functions *H_z,p,r_* describe the activation kinetics for the concentrations of ECM molecules, proteases and ECM receptors, respectively. We approximate these functions with a two-level sigmoid function of the following form:


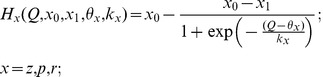
(11)

In this equation, *x_0_* and *x_1_* are the asymptotic levels with *Q→*±*∞*, respectively, *θ_x_* is the activation midpoint, and *k_x_* is the inverse slope of the activation curve. Following phenomenological observations [1], [7], [16], we choose the parameters such that the curves have the shapes illustrated in Fig. 4 A.

**Figure 10:**
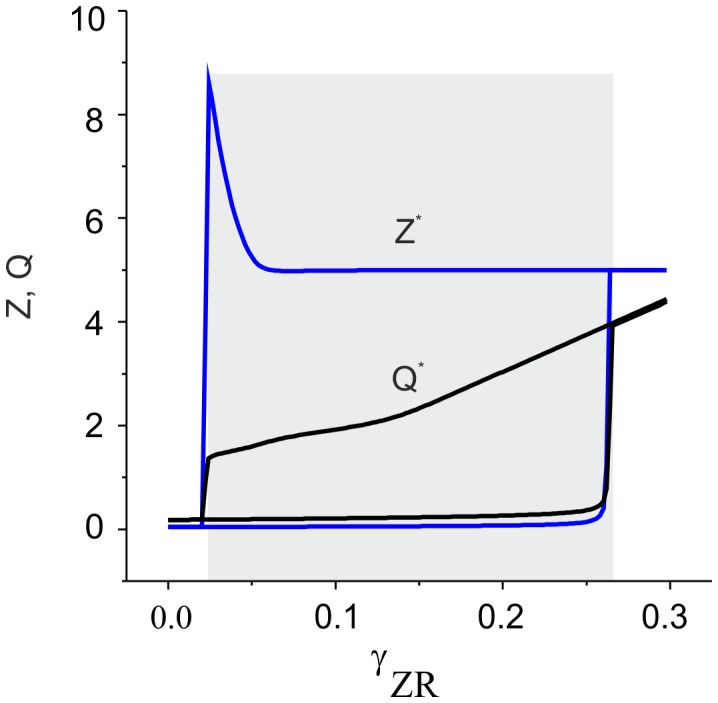
Bistability induced by the ECM receptors regulation cascade. Black and blue curves show stable steady state activity levels and ECM concentrations, depending on the feedback gain. The gray area illustrates bistability. Parameter values: *z_0_ = p_0_ = 0, z_1_ = p_1_ = 1, θ_z_ = 1,k_z_ = 0.15, θ_p_ = 1.5,k_p_ = 0.05, α_z_* = 0.001 *msec^−1^*, *β_z_* = 0.01 *msec^−1^*, *α_p_* = 0.001 *msec^−1^*, *α_3_* = 0.0001 *msec^−1^*, *α_4_* = 0.001 *msec^−1^, I_0_ = 4.5 µA/cm^2^,b_0_ = 6, Q_0_ = 0.18,* and *k_b_ = 0.4762*.

Parameters *α_x_ (x = z,p,r)* define the rate of spontaneous degradation of ECM concentration, proteases and ECM receptors, respectively. Parameters *β_x_ (x = z,p,r)* describe the activation rate of the corresponding variables. Note that the values of rate constants also adjust the time scales between fast spiking defined by Eqs. (1)–(3) and slow ECM feedback in Eq. (9). The latter is assumed to be at the time scale of hours or days, hence the rates should be in the range of 10^−7^–10^−8^ sec^−1^. Here, to speed up numerical illustrations, we used much higher values (10^−3^ msec^−1^) assuming that the time scale can be arbitrary adjusted by tuning the parameters. However, this is not critical because the longer time scale, the better predictions are for the mathematical model.

**Figure 11:**
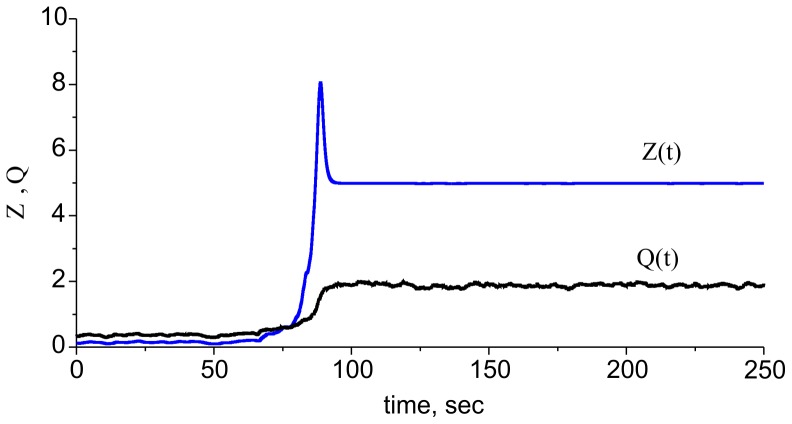
Dynamics of the ECM receptor regulation cascade in the spiking model (1)–(6), (9), and (10). Due to the integration of the Poisson input train, the *Q* variable begins fluctuating near the *Q_∞_* value (see Eq. (7) in Methods). This figure illustrates a spontaneous transition from the lower to higher level state. Parameter values: *z_0_ = p_0_ = 0, z_1_ = p_1_ = 1, θ_z_ = 1,k_z_ = 0.15, θ_p_ = 1.5,k_p_ = 0.05, k_q_* = 0.01, *γ_p_* = 0.1, *I_o_ = 4.5 µA/cm^2^, b_0_ = 6.5, γ_Z_ = 0.0, γ_ZR_ = 0.065, α_q_* = 0.0001 *msec^−1^*, *β_q_* = 0.01 *msec^−1^*, *α_z_* = 0.001 *msec^−1^*, *β_z_* = 0.01 *msec^−1^*, *α_p_* = 0.001 *msec^−1^*, *β_p_* = 0.01 *msec^−1^* and *f_input_ = 0.2 kHz*.

Eqs. (10) implement two distinct methods for modifying neuronal dynamics through feedback influences of the ECM. The first changes excitability levels by changing the effective excitation threshold [7]. We model this effect in the simplest form by changing the depolarization level necessary to elicit an action potential. The feedback gain is described by parameter *γ_Z_*. The second loop modifies synaptic weights, depending on product *ZR* with gain *γ_ZR_* and results in the re-scaling of the EPSC distribution (see Methods, Fig. 1A) and therefore the potentiation or depression of synaptic inputs. Generally, as we have already mentioned, the impact of this feedback can be bidirectional.

**Figure 12:**
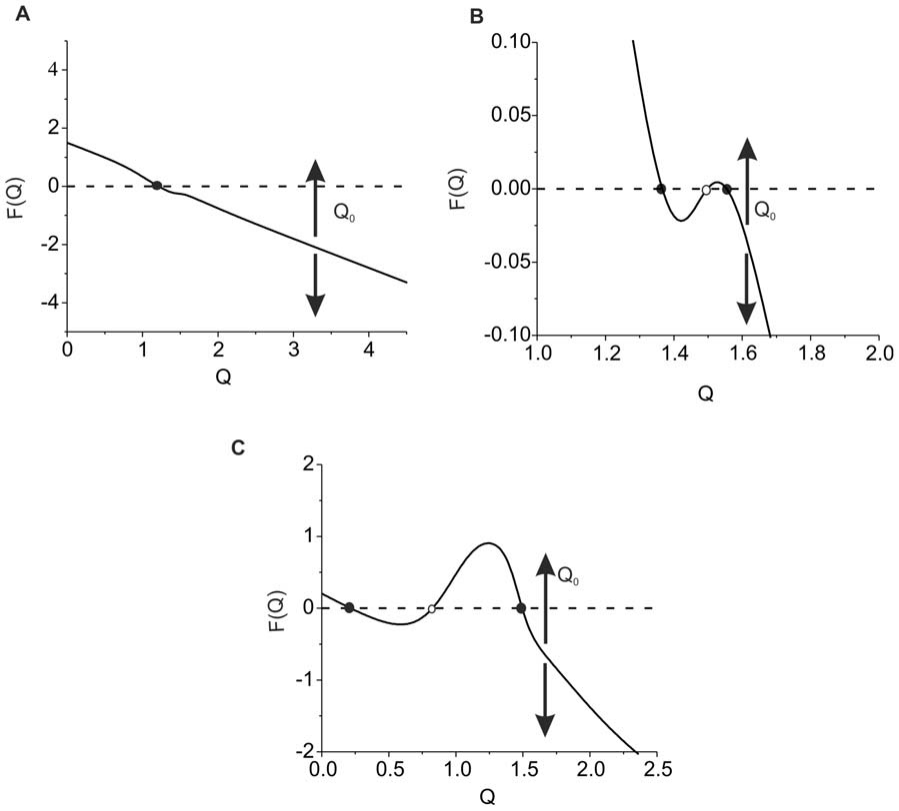
Interplay of the two regulation mechanisms balancing average activity. Steady state activity levels are defined by zeros of function *F(Q)*. Parameter values: *z_0_ = p_0_ = 0, z_1_ = p_1_ = 1, θ_z_ = 1, k_z_ = 0.15, θ_p_ = 1.5, k_p_ = 0.05, k_q_* = 0.01, *α_q_* = 0.0001 *msec^−1^*, *β_q_* = 0.01 *msec^−1^*, *α_z_* = 0.001 *msec^−1^*, *β_z_* = 0.01 *msec^−1^*, *α_p_* = 0.001 *msec^−1^*, *β_p_* = 0.01 *msec^−1^*, *γ_p_* = 0.1, *I_0_ = 4.5 µA/cm^2^, b_0_ = 6, k_I_ = 0.6625,* and *k_b_ = 0.4762.* **A.** Monotonic regulation for rather low feedback gains, *γ_Z_ = 0.03, γ_ZR_ = 0.01, Q_0_ = 1.5*. **B.** Bistability for high input frequency, *γ_Z_ = 0.04, γ_ZR_ = 0.01,* and *Q_0_ = 1.95*. **C.** Bistability for low input frequency, *γ_Z_ = 0.1, γ_ZR_ = 0.1,* and *Q_0_ = 0.2*.

The feedback mediated by proteases is implemented in Eqs. (9) as a nonlinear relaxation of the ECM concentration controlled by gain parameter *γ_P_*.

### 2.3. Steady State Activation; Leveling Average Activity

First, let us consider the steady state values (*Z_∞_*, *P_∞_*, *R_∞_*) of the species involved in model (9) for different levels of average activity *Q*, which are determined by the activation functions as follows:


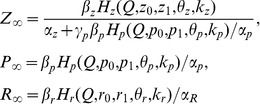
(12)

Figure 4 B illustrates the dependence of the steady state values on *Q*.

**Figure 13:**
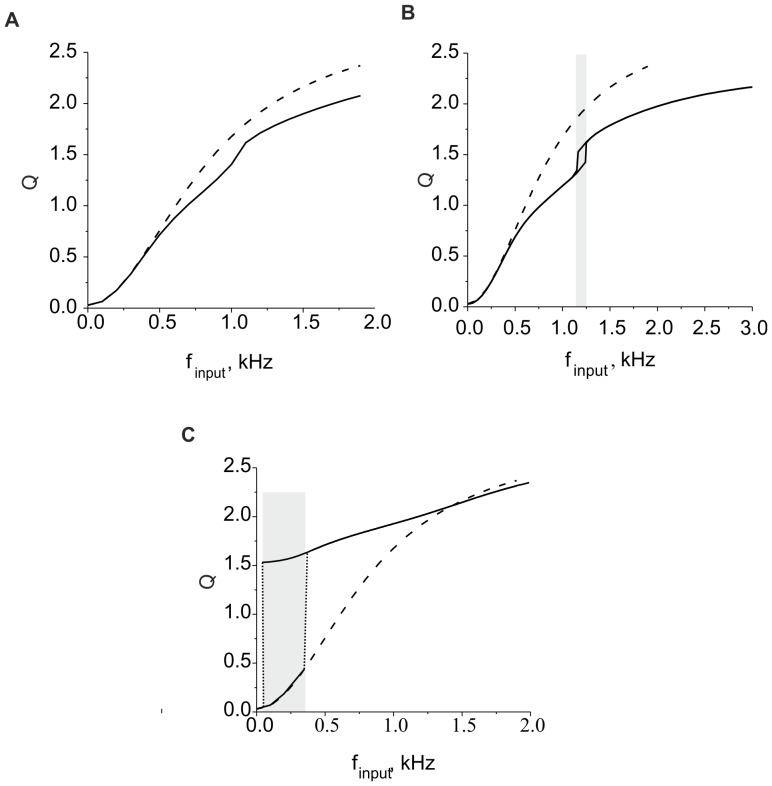
Steady state average activity depending on the frequency of synaptic input (solid curve). Dashed curve shows the activity curve without feedback. Parameter values: *z_0_ = p_0_ = 0, z_1_ = p_1_ = 1, θ_z_ = 1,k_z_ = 0.15, θ_p_ = 1.5,k_p_ = 0.05, k_q_* = 0.01, *α_q_* = 0.0001 *msec^−1^*, *β_q_* = 0.01 *msec^−1^*, *α_z_* = 0.001 *msec^−1^*, *β_z_* = 0.01 *msec^−1^*, *α_p_* = 0.001 *msec^−1^*, *β_p_* = 0.01 *msec^−1^*, *I_0_ = 4.5 µA/cm^2^, b_0_ = 6, k_I_ = 0.6625,* and *k_b_ = 0.4762.* **A.** Monotonic regulation for low enough feedback gains, *γ_Z_ = 0.03* and *γ_ZR_ = 0.01*. **B.** Bistability for high input frequency, *γ_Z_ = 0.04* and *γ_ZR_ = 0.01*. **C.** Bistability for low input frequency, *γ_Z_ = 0.1* and *γ_ZR_ = 0.1*.

Curves for *P_∞_ (Q)* and *R_∞_(Q)* show that there are two stationary levels of proteases and ECM receptor concentrations. The proteases become activated if the activity exceeds a certain critical level. This activation acts through feedback on the ECM concentration, which leads to higher degradation rates. The ECM concentration therefore has a peak. The ECM receptor expression rate is higher for lower activity ranges [16]. Thus, function *Z_∞_R_∞_ (Q)* also has a maximum for a certain activity level.

Such behavior of the steady state curves will induce the activity regulation cascades described below in sections *2.3.1 and 2.3.2*.

#### 2.3.1. ECM – protease regulation cascade

An increase in the average firing rate results in ECM production. Next, the feedback lowering the excitation threshold tends to decrease the activity level providing depressive feedback. Due to the presence of the peak in the *Z_∞_(Q)* function, the cooperative action of ECM and proteases results in an increase of activity if the proteases are activated.

**Figure 14:**
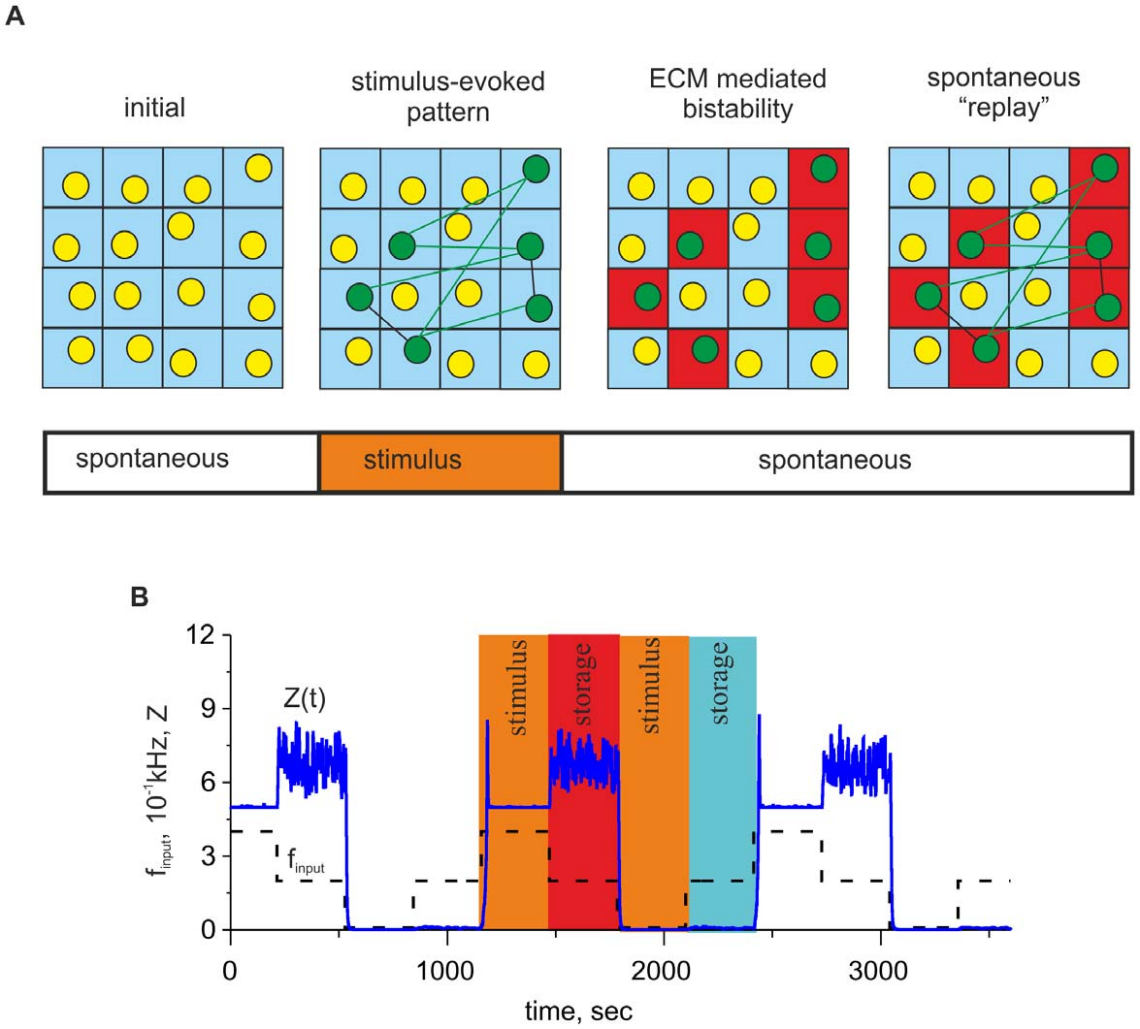
Potential contribution of ECM-mediated bistability to memory consolidation. **A.** Schematic network model illustrating how the bistability may contribute to consolidation of activity routes (from left to right). Spontaneous dynamics results in the lower level of ECM concentration. Afferent stimulation generates some activity propagation routes and, hence, increases firing rate in some cells (green circles). The ECM associated with these cells is upregulated (red squares) and may sustain their activity even after the afferent stimulus terminates. Consequently, due to higher output spiking rate these neurons may facilitate replays of the activity routes and facilitate memory consolidation. **B.** Response of a network neuron associated with ECM to a bipolar stimulus (dashed curve) with *f_in0_ = 0.2 kHz* and *f_signal_ = 0.1, 0.4 kHz*. Color areas show time intervals of stimulation and spontaneous evolution at the higher (red color) and at the lower (blue color) level states. Note that there are fluctuations in *Z* after excitatory stimulation period, which are due to the feedback mediated by extracellular proteases. The amplitude of the spontaneous fluctuations is proportional to 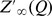. Parameter values: *z_0_ = p_0_ = 0, z_1_ = p_1_ = 1, θ_z_ = 1, k_z_ = 0.15, θ_p_ = 1.5,k_p_ = 0.05, k_q_* = 0.01, *α_q_* = 0.0001 *msec^−1^*, *β_q_* = 0.01 *msec^−1^*, *α_z_* = 0.001 *msec^−1^*, *β_z_* = 0.01 *msec^−1^*, *α_p_* = 0.001 *msec^−1^*, *β_p_* = 0.01 *msec^−1^*, *I_0_ = 4.5 µA/cm^2^, b_0_ = 6, γ_P_ = 0.1, γ_ZR_ = 0.03* and *γ_Z_ = 0.0*.

Let us estimate the conditions in which a balance is achieved. It follows from the membrane excitability model (1)–(6) that the average activity level corresponds with certain values of the depolarization level controlled by current *I_th_*, as illustrated in Fig. 5. Increasing *I_th_* leads to hyperpolarization, hence larger excitatory input is needed to generate a spike, and at some level, the activity tends to die out. Note that for relatively large values of *Q,* the curves can be well fit by linear dependence in the following form:


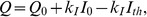
(13)

where *Q_0_* is the average activity value for threshold parameter *I_th_ = I_0_*. When the feedback is activated, *I_th_* is changed with gain *γ_Z_* depending on the ECM concentration (see Eq. (10)). Therefore, in the steady state conditions, one can define equilibrium curve *Q_a_(Z)*, i.e., the dependence of the average activity on fixed values of *Z* in the spiking model (1)–(6), as follows:


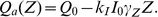
(14)

In this equation, *I_0_* and *Q_0_* define the activity level in the model without feedback. Using (12) and (14), one can find that the steady state activity balance is defined by the intersection points of line *Q_a_(Z)* and curve *Z = Z_∞_(Q)*. Surprisingly, this balance condition may lead to interesting outcomes in the *ECM –* protease regulation cascade. Figure 6 shows three principle arrangements of equilibrium curves *Q_a_(Z)* and *Z_∞_(Q).* Different cases may be realized for different input frequencies *f_input_* and different values of feedback gain *γ_Z_*.

The equilibrium curves may have from one to three intersection points, depending on the initial activity level. As one may expect, the activation of feedback leads to the suppression of the average activity and decrease of ECM concentration (Fig. 6 A). However, if the activity is sufficiently high, e.g., when the proteases are activated, the feedback would promote a growth of the ECM concentration (Fig. 6 B). Higher gains may lead to a bistability effect; i.e., they may lead to the evolution of steady states with low and high ECM concentrations and thus different activity levels (Fig. 6 C), depending on initial conditions.

Note that conditions (12) and (14) define the existence of the steady state solutions only. These conditions’ stability must be analyzed in terms of the original Eqs. (1)–(6), (9), and (10). Because spikes in Eqs. (1)–(5) are generated on a much faster timescale relative to variables *Q, P, Z,* and *R* and feedback (10) is instantaneous, we may approximate the dynamics in a quasistationary limit. In numerical simulations, we found that the average activity *Q* that emerged from the spiking dynamics of Eqs. (1)–(6) converged to *Q_a_(Z)* under any initial conditions. Accordingly, the average dynamics of the *Q* variable can be approximated as the relaxation of *Q* to its equilibrium curve *Q_a_(Z).* Similarly, according to Eqs. (9) and (10), the dynamics of the *Z* variable can be described as its relaxation to equilibrium curve *Z_∞_(Q)*. Thus, the average dynamics can be described by the following equations:


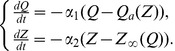
(15)

Here, we assumed that variables *Q* and *Z* converge to their equilibrium curves with relaxation rates *α_1_* and *α_2_*. For simplicity, we assume that the rates are constant and do not depend on variables *Q, Z, P* or *R*. It is easy to find that the stability of a fixed point of Eqs. (15), (Q*,Z*), is defined by the eigenvalues:


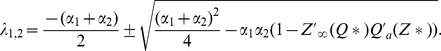


Using Eq. (14), the stability condition can be written as follows:


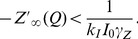
(16)

Thus, according to the curves shown in Fig. 6, the fixed point defining the equilibrium ECM concentration is always stable, with the only exception being when it lies in the middle branch of the *Z_∞_(Q)* curve, as shown in Fig. 6 C.

Figure 7 illustrates the ECM-protease regulation cascade for different feedback gains obtained in numerical simulations using Eqs. (9) and (14). The average activity is given by the two curves breaking apart in the region of bistability. As expected for depressive feedback, the curves are decreasing functions of the gain. However, the ECM concentration shows a bidirectional regulation behavior; it may increase due to the feedback splitting on two stable curves in the bistable region, and it may decrease for higher gain values. The presence of two branches in the activity curves indicates the hysteresis effect typical for bistable dynamical systems when the dynamics are different for increasing and decreasing gains.

In a simulation of the original model (1)–(6), (9), and (10), the average activity *Q* induced by the Poisson synaptic input always has small fluctuations near the limit value *Q_∞_* (see Eq. (7) and Fig. 2 in Methods). In the single fixed-point modes, these fluctuations are projected to the ECM concentration that oscillates proportionally to *dZ_∞_(Q)/dQ*. However, in the bistable mode, the ECM – protease feedback cascade leads to spontaneous transitions from one state (high activity – low ECM) to the other (low activity – high ECM), as illustrated in Fig. 8.

#### 2.3.2. ECM receptor regulation cascade

Next, we consider the feedback loop mediated by ECM receptors. The dynamics of the receptors are described by the third equation in the system (9), leading to equilibrium concentration *R_∞_(Q)* (Fig. 4 B). The impact of the ECM on modulating the synaptic weight distribution (the second equation in (10)) is determined by equilibrium curve *Z_∞_R_∞_*, as shown in Fig. 4 B. The curve has a peak for the activity interval corresponding with the maximum rate of ECM receptor expression. The presence of this peak determines the key outcome of the ECM receptor regulation cascade. If the activity is sufficiently high, the ECM impact is defined by saturated values of ECM and ECM receptor concentration and almost independent of changes in activity (the flat interval of *Z_∞_R_∞_* curve to the right of its maximum in Fig. 4 B). However, if the activity is decreasing (for instance, due to a decrease in either input activity or ECM-proteases feedback), the ECM impact increases to its maximum value at the peak of the *Z_∞_R_∞_* curve, which tends to stabilize the activity level. Interestingly, for the lower activities (left of the peak), the ECM regulation may act in the opposite direction by decreasing activity because the *Z_∞_R_∞_* values become lower than the initial level; therefore, the activity decreases due to the depression of synaptic input.

Let us construct a mathematical model of the ECM receptor regulation cascade. First, we assume that the gain of ECM feedback *γ_Z_* is negligible, and a neuron is regulated by changes in its synaptic input controlled by gain *γ_ZR_*. In calculating the dependence of average activity on the strength of synaptic input (see Methods, Eqs. (1)–(6)), we find that the *Q* variable converges to curve *Q = Q_b_(b),* as illustrated in Fig. 9A, for different values of the input frequency. Taking into account Eqs. (10), we obtain conditions defining the equilibrium activity as follows:


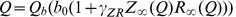
(17)

Figure 9B illustrates the solutions of Eq. (17) in phase plane *(Q,ZR)*. These solutions are given by the intersection points of curves *Q = Q_b_(ZR)* and *ZR* = *Z_∞_R_∞_(Q).* Similar to the case of the ECM-protease regulation cascade, the ECM receptor impact may be different, depending on the values of input frequency that determine *Q_0_ = Q_b_(ZR = 0)* and the feedback gain *γ_ZR_* that controls the slope of the *Q_b_* curves. If there is one intersection point, the level of equilibrium activity will be higher than in conditions without the feedback. Note, however, that the feedback may induce bistability when there are three intersection points between the equilibrium curves (Fig. 9 B).

The steady state stability problem must be addressed in terms of the original model (1)–(6), (9), and (10) and represents a notably complicated task. In this study, we reduce this problem to a linear approximation model in the following manner: let us approximate function *Q_b_(ZR)* by a linear fit as follows:


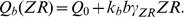
(18)

Accordingly, similar to Eq. (15), for local perturbations, we can write the following equations:


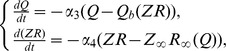
(19)

with relaxation rates *α_3_* and *α_4_*, which do not depend on variables *Q, Z, P* or *R* in the approximation of linear relaxation. Thus, the stability of the steady state (Q*,(ZR)*) is defined by a pair of eigenvalues:


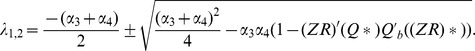


Thus, the steady state is stable if the following inequality is satisfied as follows:


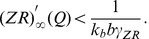
(20)

It follows from Fig. 9B that when there are three intersection points, i.e., three steady state activity levels, the middle point is of saddle type, “repelling” the system to one of the two stable states. To confirm this prediction, we simulate Eqs. (9,18) numerically. Figure 10 illustrates the bistability induced by the ECM receptor regulation for sufficiently low initial activity (small input frequency and/or low synaptic input). As predicted, the response curves have two branches corresponding with low and high values of the outcome level of the average activity and ECM concentration.

Next, we determined bistability effects in terms of the original spiking model (1)–(6), (9), and (10). Figure 11 illustrates spontaneous transitions from one stable state to another induced by the fluctuations of average activity under Poisson train stimulation. In this example, the higher-level steady state is located to the right of the peak of the *ZR* equilibrium curve (Fig. 9B). This peak is clearly observed in Fig. 11 in the transition of the ECM concentration to its higher-level state, which corresponds with the activation of proteases that tends to decrease the ECM level.

#### 2.3.3. Balancing the average activity with the two feedbacks

Let us consider the dynamics of the complete circuit (Fig. 3), i.e., when the two feedback mechanisms are activated. According to feedback Eqs. (10), the average activity level is defined by the value of two key parameters *I_th_* and *b,* which become slowly variable (relative to the millisecond timescale of the spiking response), due to changes in *Z* and *ZR*. Then, for variable *Q*, one can write that.


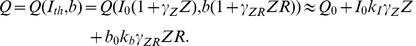
(21)

In this equation, for illustration, we assumed that *Q* depends linearly on both *Z* and *ZR* (i.e., a linear approximation of the nonlinear dependences, as shown Figs. 5,9A). Constants *I_0_,b_0_* are defined by the excitability of the neuron, and *Q_0_* depends on the input frequency. To find the steady state level of the average activity, we assume that *Z* and *R* tend to their equilibrium functions *Z_∞_(Q)* and *R_∞_(Q)*, respectively. Thus, the steady state values correspond to the zeros of the following function:


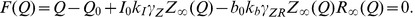
(22)

Figure 12 illustrates three possible solution types of Eq. (22) depending on feedback gains *γ_Z_* and *γ_ZR_*. When function *F(Q)* is monotonic, there is only one level of the average activity changing with *Q_0_* for different input frequencies (Fig. 12 A). This case is realized when both feedbacks have sufficiently low values. Changing *Q_0_* corresponds with the shift of the *F(Q)* curve, as indicated by arrows, and thus changes in the average activity level. Increasing the value of the feedbacks may lead to bistability in the response activity, as illustrated in Fig. 12 (B and C). When the “ECM - proteases” regulation cascade prevails, bistability appears for high inputs (Fig. 12 B). The interval of bistability is defined by the activation of proteases (activation function *H_p_*). For sufficiently low input frequencies (Fig. 12 C) bistability corresponds with the contribution of the ECM receptor feedback, as predicted by Eqs. (19).

Finally, we derive a general characteristic of the ECM regulation model by analyzing its input-output frequency dependence. Note that the average activity, as described in the Methods, is proportional to the output frequency of the neuron (Fig. 2A) and depends on the input frequency according to a logistic law (Eq. (8), Fig. 2B). For sufficiently low feedback gains (Fig. 12A), the response is monotonic, as illustrated in Fig. 13A. The response curve goes below the dashed curve, corresponding with the model without feedback. Therefore, as one may expect, the ECM regulation tends to decrease the response activity to increasing intensities of synaptic input and provides homeostatic regulation. Surprisingly, this regulation may have a bistable character. Figure 13B illustrates bistability realized for high activity. This bistability has a narrow interval but may eventually result in an increase in the response curve associated with the activation of proteases, as mentioned earlier. Figure 13C illustrates bistability for higher gains of ECM receptor feedback which occurs in a relatively broad range of input frequencies. The upper branch has a flat shape, which indicates the spiking rate balance for both high and low activity. However, for low input frequencies, the activity may decrease to the lower branch of the response curve.

## Discussion

In summary, we have developed a mathematical model of ECM-mediated regulation of neuronal activity. The model reflects key experimental observations regarding the influence of the ECM on neuronal signaling. The model comprises the following features: (i) expression of ECM and ECM degrading enzymes is controlled by the neuronal activity, (ii) fluctuation of the firing threshold depends on the ECM concentration; (iii) there is modulation of synaptic weights due to ECM receptor signaling in dendritic spines. Mathematically, the model is expressed by a set of eight ordinary differential equations. Because the ECM dynamics are much slower than spiking dynamics, we have investigated the model analytically in a quasistationary approximation. This analysis revealed conditions under which the ECM may act as an effective regulation factor for sustaining homeostatic balance in neuronal firing. The ECM prevents neuron over-excitation if the average activity becomes too high, and in contrast, the ECM may help the neuron sustain its firing rate in cases of a dramatic decrease in synaptic input. These two mechanisms are expected by the model construction because the increase of the excitation threshold immediately reduces the firing rate, and the expression of ECM receptors due to lowering activity facilitates synaptic transmission. Unexpectedly, we found that the interplay between the feedbacks may induce bistability and coexistence of two stable firing rates in homeostatic conditions; this finding suggests that under the certain perturbations (for example, transient increases or decreases in input firing rates), the neuron may jump between the lower and higher spiking rate levels.

Note that the bistability is a purely nonlinear effect. It has been reported in several papers on neurodynamics that bistability may naturally emerge from cell membrane dynamics [18]–[19]. In particular, bistability is associated with complex membrane potential dynamics [19], resonant interactions and signal processing in dendritic trees [20], generation of episodic discharges in inhibitory interneurons [21] and other mechanisms. However, the most interesting point for computational considerations is that bistability is associated with memory (for example, see the Hopfield network paradigm [22]). This finding is in line with the hypothesis that the ECM “may contain memory traces of local neural network activity”, as recently proposed by Dityatev and Rusakov [14]. The key point is that due to ECM influence, the neuron is capable of sustaining two different levels of output spiking rate, depending on its previous activity. In terms of our model, perhaps the simplest treatment of how the ECM could be utilized to generate memory traces is the following.

Let us consider, for example, a network of neurons in a state of spontaneous activity when the average input frequency is in the interval of bistability (for example, see Fig. 13C). Starting from rather low initial concentrations, the model converges to the low steady state of activity (hence, a low ECM concentration), which corresponds with the lower branch of the bifurcation diagram (Fig. 13C). Let us assume that the network receives afferent stimulation, and the signal circulates along synaptic pathways for a long duration. This results in a non-uniform distribution of synaptic inputs from different neurons, which reflects the spatial organization of the corresponding signaling pathways. Thus, different network neurons will receive different input frequencies *f_input_ = f_in0_+f_signal_*. The neurons receiving more intensive stimulation may change their steady states to the higher state, as illustrated in Fig. 14 A. Finally, when the afferent stimulus terminates, the system may remain at the upper firing state for a long duration because it represents a stable configuration for spontaneous frequency *f_in0_*. In particular, such dynamics may be a substrate for the generation of replays to consolidate memory after the stimulus because the neurons at the higher firing state memorize the network sites activated by the stimulus; i.e., they represent memory traces. Figure 14B illustrates the model dynamics with *f_signal_(t)* taken in the form of a low frequency bipolar signal relative to spontaneous level *f_in0_*. Excitatory and inhibitory signals induce transitions to the higher and lower ECM concentrations, respectively, “memorizing” the level of activity generated by the neuron and determining its later steady state of activity. Note that the memorizing effect is local and specific to particular neuron involved in a network implementing memory function. Note also that bistability enhances major model effect of homeostatic spiking rate adaptation. The neuron associated with ECM is capable to sustain two distinct activity levels depending on the context of neuron operation within a network.

These qualitative considerations remain to be verified using computer simulations of the activity in “realistic” neuronal networks of ECM-associated neurons after further elaboration of the principles governing: (i) secretion of diverse ECM molecules in distinct subcellular compartments, such as (peri)synaptic region and axon initial segment; (ii) activation of diverse extracellular proteases, such as tissue plasminogen activator, plasmin, matrix metalloproteinase-9, neuropsin and neurotrypsin; (iii) the activities of these proteases on specific ECM substrates; and (iv) ECM-mediated regulation of synaptic transmission and excitability in inhibitory versus excitatory neurons. These elaborated models can provide an insight into the ECM-mediated role in epileptogenesis [23], [24] and memory deficits associated with ECM remodeling in neurodegenerative disorders [25].
